# The Dual Roles of Lamin A/C in Macrophage Mechanotransduction

**DOI:** 10.1111/cpr.13794

**Published:** 2024-12-22

**Authors:** Yao Wang, Sabine Ruf, Lei Wang, Thomas Heimerl, Gert Bange, Sabine Groeger

**Affiliations:** ^1^ Department of Orthodontics, Faculty of Medicine Justus Liebig University Giessen Germany; ^2^ Department of Oral and Maxillofacial Surgery, Affiliated Stomatological Hospital Southwest Medical University Luzhou P. R. China; ^3^ Center for Synthetic Microbiology (SYNMIKRO) Philipps‐University Marburg Marburg Germany

**Keywords:** compressive force, lamin A/C, LINC complex, macrophage, mechanotransduction, YAP1

## Abstract

Cellular mechanotransduction is a complex physiological process that integrates alterations in the external environment with cellular behaviours. In recent years, the role of the nucleus in mechanotransduction has gathered increased attention. Our research investigated the involvement of lamin A/C, a component of the nuclear envelope, in the mechanotransduction of macrophages under compressive force. We discovered that hydrostatic compressive force induces heterochromatin formation, decreases SUN1/SUN2 levels, and transiently downregulates lamin A/C. Notably, downregulated lamin A/C increased nuclear permeability to yes‐associated protein 1 (YAP1), thereby amplifying certain effects of force, such as inflammation induction and proliferation inhibition. Additionally, lamin A/C deficiency detached the linker of nucleoskeleton and cytoskeleton (LINC) complex from nuclear envelope, consequently reducing force‐induced DNA damage and IRF4 expression. In summary, lamin A/C exerted dual effects on macrophage responses to mechanical compression, promoting certain outcomes while inhibiting others. It operated through two distinct mechanisms: enhancing nuclear permeability and impairing intracellular mechanotransmission. The results of this study support the understanding of the mechanisms of intracellular mechanotransduction and may assist in identifying potential therapeutic targets for mechanotransduction‐related diseases.

## Graphical abstract

Compressive force can inhibit the expression of lamin A/C, SUN1 and SUN2 in macrophages. Lamin A deficiency may participate in mechanotransduction within macrophages through two distinct mechanisms.

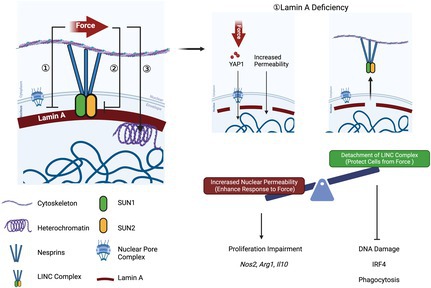

## 1 Introduction

Macrophages, essential innate immune cells, are ubiquitous in all tissues and body compartments [1]. Until now, most studies have primarily focused on biochemical stimulation applied to macrophages. However, recent research has revealed that macrophages can also respond to various forms of biomechanical stimulation, such as cyclic tensile force [2], orthodontic compression [3], spatial confinement [4], and matrix stiffness [5]. The mechanical activation of macrophages plays a pivotal role in various physiological processes and pathological conditions, including cancer [6], steatohepatitis [7], orthodontic tooth movement [8], osteogenesis [9], pulmonary inflammation [10] and atherosclerosis [11]. A prerequisite for cells to respond to external mechanical forces is their ability to convert these mechanical signals into intracellular biochemical signals. Elucidating this process, known as mechanotransduction, remains a challenge because it involves switching signals between multiple forms. It has been reported that the nucleus plays an important role in mechanotransduction [12, 13]. Although this response, primarily in the form of changes in gene transcription, occurs at a slower time scale compared to the mechanotransduction events in the cytoplasm, which are independent of the nucleus, it is crucial for the long‐term cellular adaptation to mechanical forces [14]. Our previous study demonstrated that hydrostatic compression promoted histone modification, eventually causing macrophage polarisation [15]. This study aimed to elucidate further upstream mechanisms, specifically how external compression signals are transmitted to the nucleus, a structure deep within the cell.

The nuclear lamina, a protein network approximately 15 nm thick located beneath the inner surface of the nuclear envelope, consists of four distinct types of intermediate filaments in mammalian cells: lamin A, B1, B2 and C [16, 17, 18]. Lamin A and its splice variant lamin C, both encoded by the *LMNA* gene, have high similarities in their primary sequences, so they have mostly been studied together as lamin A/C [19]. Lamin A/C interacts with transcription factors, the nuclear pore complex (NPC) and chromatin and is believed to be associated with nuclear stiffness, cellular migration and differentiation [17, 20]. Recently, lamin A/C was found to respond to mechanical stimulation [20, 21, 22]. Specifically, lamin A/C is linked to the cytoskeleton and responds to external mechanical cues, such as shear force or extracellular matrix stiffness, which can affect lamin A/C protein levels or molecular conformation [20, 23]. Clinically, deficient lamin A/C is linked to various pathological changes, including premature aging, cardiomyopathy, lipodystrophy, muscular dystrophy, microcephaly, epilepsy, mandibuloacral dysplasia type A and axonal peripheral neuropathy [24, 25]. These observations support the involvement of lamin A/C in cellular mechanotransduction and related diseases. However, the role of lamin A/C in mechanotransduction under hydrostatic compression, a type of mechanical stimulus found in tissues such as bones, muscles, the bladder, the interior of the eyeball and periodontal ligaments, remains unclear.

Lamin A/C connects to the cytoskeleton via the linker of nucleoskeleton and cytoskeleton (LINC) complex [20, 26]. The LINC complex is a protein complex expressed on the nuclear envelope, consisting of the outer nuclear membrane Klarsicht/ANC‐1/Syne‐1 homology (KASH) domain proteins (such as nesprin family members) and the inner nuclear membrane (INM) Sad1/UNC‐84 (SUN) domain proteins (such as SUN1 and SUN2) [27]. The N‐terminus of KASH domain proteins interacts with cytoskeleton [27]. SUN proteins are anchored in the INM by at least one transmembrane segment, exposing their N termini to the nucleoplasm and connecting with nuclear binding partners [28]. Taking SUN2 as an example, the SUN2 protein trimer passes through the INM and is connected to the nuclear lamina via a mechanosensitive domain [29]. Through this physical connection, lamin A/C can directly receive external mechanical cues. Additionally, lamin A/C also interfaces with chromatin, participating in diverse cell signalling pathways that influence fundamental cellular processes such as proliferation, migration, genome organisation and DNA repair [23]. Hence, we hypothesised that lamin A/C mediates macrophage mechanotransduction as a nuclear mechanical sensor, bridging external mechanical stimuli with gene transcription.

## 2 Materials and Methods

### 2.1 Cell Culture and Compressive Force Loading

**FIGURE 1:**
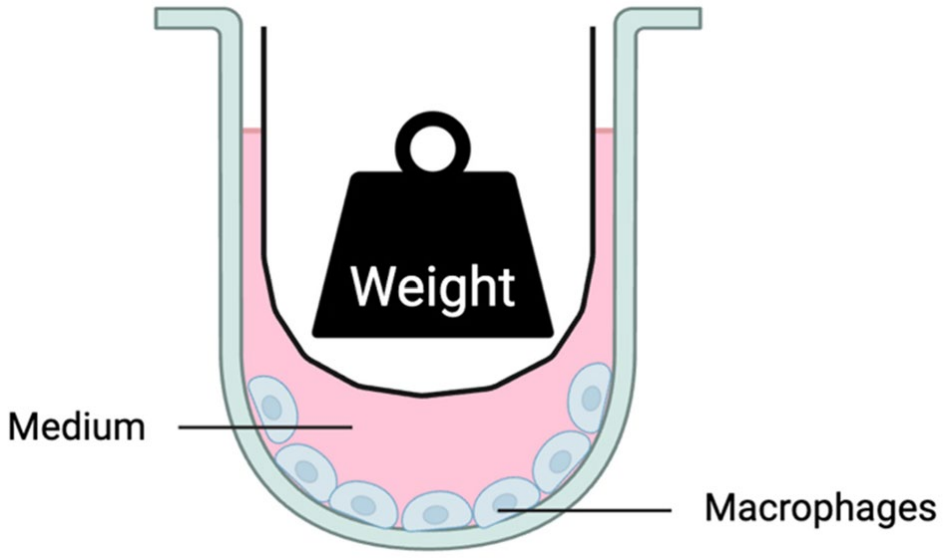
Schematic diagram illustrates the model used for hydrostatic compressive force. A weight and its container, represented as a ‘ship’, float on the medium, applying compressive force to the cells in the form of hydrostatic pressure.

The murine macrophage cell line RAW264.7 was purchased from the ATCC. Briefly, the macrophages were cultured in DMEM (41965062, Gibco) with 10% foetal bovine serum (FBS) (10270‐106, Gibco) and 1% penicillin/streptomycin (A3160502, Gibco). The cells were incubated in a humidified atmosphere of 5% CO_2_ at 37°C. The cells were seeded into 6‐well plates (657160, Greiner Bio‐one) at a density of 1 × 10^6^ cells/well 24 h before compressive force loading. The contactless force‐loading appliance utilised has been described in our previous publication [15]. Briefly, a floating weight was placed on the medium to subject macrophages to 1 g/cm^2^ of hydrostatic pressure. Cells were harvested by a scraper to operate further analysis (Figure 1).

### 2.2 Transmission Electron Microscopy (TEM) Analysis

Each of the fixed and concentrated cell suspensions (3 μL) was high‐pressure‐frozen (Wohlwend HPF Compact 02) and freeze‐substituted (AFS2, Leica). A medium based on acetone, containing 0.25% osmium tetroxide, 0.2% uranyl acetate and 5% ddH_2_O, was used. All samples were freeze‐substituted according to the following protocol: −90°C for 22 h, from −90°C to −60°C for 1 h, −60°C for 8 h, from −60°C to −30°C for 1 h, −30°C for 8 h and from −30°C to 0°C for 1 h. At 0°C, samples were washed three times with acetone before a 1:1 mixture of Epon 812 substitute resin (Fluka) and acetone was applied at room temperature (RT) for 2 h. The 1:1 mixture was substituted with a 2:1 mixture overnight, followed by fresh pure resin the next day for 2 h to impregnate the samples. After another substitution with fresh Epon, samples were polymerised at 60°C for 2 days. The polymerised Epon blocks were then trimmed with razor blades and cut into 50‐nm ultrathin sections using an ultramicrotome (UC7, Leica) and a diamond knife (Diatome). Sections were applied onto 100 mesh copper grids coated with pioloform. For additional contrast, mounted sections (labelled or unlabelled) were post‐stained with 2% uranyl acetate for 20–30 min and subsequently with lead citrate for another 1–2 min. The sections were finally analysed and imaged using a JEOL JEM‐2100 transmission electron microscope operated at 120 kV and equipped with a TVIPS 2 k × 2 k F214 fast‐scan CCD camera.

### 2.3 Click‐iT EdU Proliferating Assay

The proliferation assay was conducted using the Click‐iT EdU Flow Cytometry Assay Kit (C10425, ThermoFisher) according to the manufacturer's instructions. Briefly, cells were seeded into 6‐well plates at a density of 2 × 10^5^ cells per well. Following a 2‐h incubation with Click‐iT EdU, cells were harvested, resuspended in PBS containing 1% BSA and subsequently fixed and permeabilised. The cells were then incubated with the Click‐iT reaction cocktail for 30 min at RT, shielded from light. Finally, cell proliferation was assessed on a flow cytometer (BD Biosciences, USA), and data analysis was performed using FlowJo software (TreeStar, USA).

### 2.4 Cell Viability Assay

Cell viability was assessed using the Cell Counting Kit‐8 (CCK‐) assay according to the manufacturer's instructions (HY‐K0301, MedChemExpress). Briefly, RAW264.7 cells were seeded onto glass slides in 6‐well plates at a density of 3 × 10^5^ cells per well and allowed to adhere overnight. The cells were then treated with a compressive force of 1 g/cm^2^, and the glass slides with the cells were subsequently transferred into 96‐well plates. Following treatment, 10 μL of CCK‐8 solution was added to each well, and the plates were incubated at 37°C for 1–4 h. Absorbance was measured at 450 nm using a microplate reader. The relative cell viability was calculated as a percentage of the control group.

### 2.5 Small Interfering RNA Oligonucleotides

The siRNAs targeting mouse *LMNA* (the gene coding lamin A/C) (SI01090803), negative control (NC siRNA) (1027280) and cell death control (SI04939025) were purchased from QIAGEN (Germany). Transfection mixture was formulated of 1.2 μL of *siLMNA* oligonucleotides, 12 μL of HiPerFect@ Transfection Reagent (301705, QIAGEN) and 100 μL of serum‐free medium. The mixed solution was incubated at RT for 10 min before being added to 2.3 mL of complete medium in a 6‐well plate. Meanwhile, macrophages were seeded in a density of 6 × 10^5^ cells/well. At least 24 h after transfection, 1 g/cm^2^ of compressive force was loaded as stimulus.

### 2.6 Plasmid Construction and Transfection

A custom‐synthesised siRNA‐resistant mutated LMNA gene (sR *LMNA*) was obtained from Invitrogen, which was inserted into the vector pcDNA3.1+ Hygro_A069 downstream of the CMV promoter. To confer resistance to siRNA designed to target the wild‐type LMNA sequence, ‘TTCCCACCGAAGTTCACCCTAAAG’, a synonymous mutation—replacing this sequence with ‘TTTCCCCCCAAATTTACATTAAAA’—was introduced in the siRNA target region and verified *via* Sanger sequencing. The plasmid DNA was purified from transformed bacteria and concentration‐determined by UV spectroscopy. The final construct was verified by sequencing.

### 2.7 Quantitative Real‐Time Reverse Transcriptase‐Polymerase Chain Reaction (qRT‐PCR)

The total RNA was isolated using the ReliaPrep RNA Miniprep Systems (z6011, Promega). RNA concentrations were measured at 260 nm using a spectrophotometer (Nanodrop2000, Thermo Scientific). cDNA was synthesised from 1.0 μg of total RNA using the Verso cDNA Synthesis Kit (AB1453B, Thermo Fisher) and performed on a CFX96TM System Cycler (Bio‐Rad).

The SsoAdvancedTM Universal SYBR@ Green Supermix (1723271, Bio‐Rad) was used in each reaction setup. The primers employed were mouse LMNA (QT00143213, Qiagen), inducible nitric oxide synthases (QT01547980, Qiagen), arginase 1 (QT00134288, Qiagen), IL‐10 (QT00106169, Qiagen), SUN1 (QT00139223, Qiagen) and SUN2 (QT00138530, Qiagen). *β*‐Actin (QT00095242, Qiagen) was used as housekeeping gene. Results were analysed using the Bio‐Rad CFX Manager 3.1 software.

### 2.8 Protein Extraction and Western Blotting Analysis

RIPA buffer (89901, Thermo Scientific) supplemented with a 3% protease inhibitor (78442, Thermo Scientific) was used for protein extraction. Protein concentrations were measured using PierceTM BCA Protein Assay Kit (23225, Thermo Scientific) on a direct reading Spectrophotometer (DR/2000, HACH). Further, 20 μg of protein samples were separated by electrophoresis using 10% SDS‐PAGE gel and transferred onto a nitrocellulose membrane (1704271, Bio‐Rad). The membranes were blocked with a 5% non‐fat milk (T145.1, ROTH) in 4°C for 24 h and incubated with the primary antibodies for lamin A/C (ab133256, Abcam), interferon regulatory factor 4 (SAB4501566, Sigma), activated YAP1 (ab205270, Abcam), total YAP1 (ab56701, Abcam), SUN1 (ab103021, Abcam), SUN2 (ab124916, Abcam), phosphorylated H2A histone family member X (γH2AX) (ab81299, Abcam), *β*‐actin (ab8227, Abcam) and GAPDH (G8795, Sigma) at a concentration of 1:1000. The used secondary antibodies were polyclonal goat anti‐rabbit horseradish peroxidase (HRP) (P0448, Dako) and polyclonal goat anti‐mouse HRP (P0447, Dako) in a concentration of 1:2000. The band signals were detected with a ChemiDoc Imaging System (Bio‐Rad) utilising a Clarity Western ECL Substrate (170‐5061, Bio‐Rad).

### 2.9 Live Cell Assay

A live cell assay was performed using the LIVE/DEAD Viability/Cytotoxicity Assay Kit (L32250, Thermo Fisher). Live cells were identified by their intrinsic intracellular esterase activity, indicated by the enzymatic conversion of a nearly non‐fluorescent, cell‐permeable Calcein AM into intensely fluorescent Calcein. This compound is effectively retained within live cells, resulting in uniform and intense green fluorescence. The experimental procedures were conducted according to the manufacturer's instructions. Briefly, cells were plated in a 6‐well plate at a density of 1 × 10^5^ cells per well 12 h prior to mechanical stimulation. After applying the force, Calcein AM was added to the medium, and the cells were incubated for 30 min at RT. Subsequently, the cells were examined using an inverted phase contrast fluorescence microscope (DMI6000 B, Leica).

### 2.10 Phagocytosis Assay

Phagocytosis was assessed using the Vybrant Phagocytosis Assay Kit (V6694, Thermo Fisher Scientific) according to the manufacturer's instructions. Cells were seeded in a 6‐well plate at a density of 1 × 10^5^ cells per well and incubated overnight. Fluorescent *E. coli* BioParticles were prepared by suspending them in Hanks' Balanced Salt Solution (HBSS) and sonicating for homogeneity. Following the desired stimulation, the BioParticles were added to the wells. After a 1‐h incubation at 37°C, the wells were washed to remove any non‐internalised particles. To quench extracellular fluorescence, 100 μL of trypan blue was added. The fluorescence intensity was analysed using an inverted phase contrast fluorescence microscope (DMI6000 B, Leica) and quantified with ImageJ software (National Institutes of Health and University of Wisconsin, United States).

### 2.11 Comet Assay

The comet assay was performed following the manual of the comet assay kit (ab238544, Abcam). Briefly, cells were prepared as single cell suspensions and combined with agarose at 37°C. The agarose/cell mixture was pipetted onto a glass slide. After the samples were solidified, they were treated with lysis buffer and alkaline solution included in the kit. Electrophoresis was performed *via* tris/borate/EDTA (TBE) electrophoresis (10.8 g tris base, 5.5 g boric acid, 0.93 g EDTA disodium salt and the volume adjusted to 1 L with DI H_2_O) at 2 V/cm for 15 min. Cells were stained with DNA dye provided in the kit for 15 min and viewed by a confocal laser microscope (LSM980 Airyscan 2, Carl Zeiss) using an FITC filter.

### 2.12 Evaluation of Nuclear Permeability

The evaluation method is modified from the protocol reported by Raices and Angelo [30]. The protocol is briefly described as follows: the macrophages were seeded together with the *siLMNA* into a 6‐well plate. After 24 h, cells were rinsed with cold PBS for 2 min and incubated with buffer A (20 mM HEPES pH 7.5, 110 mM KOAc, 5 mM MgCl2, 0.25 M sucrose, and protease inhibitors) for 5 min on the ice. After then, macrophages were incubated with buffer B (20 μg/mL 500 or 65–80 kDa TRITC‐dextran, 20 mM HEPES pH 7.5, 110 mM KOAc, 5 mM sodium chloride, 2 mM MgCl_2_, 0.25 M sucrose, and protease inhibitors) for 10 min. Cells were rinsed three times with cold PBS and mounted by a fluorescent mounting medium with DAPI (ab104139, Abcam). Finally, the cells were observed under a confocal laser microscope (LSM980 Airyscan 2, Carl Zeiss) with a 590‐nm (TRITC) or 353‐nm (DAPI) laser to evaluate nuclear permeability. For quantification using the Image J software (National Institutes of Health and University of Wisconsin, United States), the DAPI colouring area was selected as the region of interest (ROI), and the ratio of the dextran‐integrated density in ROI to the entire cells was calculated as the index of permeability.

### 2.13 Immunofluorescence (IF) Staining

Macrophages were fixed with 4% paraformaldehyde (30525–89‐4, Sigma‐Aldrich) for 15 min at RT. Cells were permeabilised using 0.5% Triton X‐100 (28,313, Thermo‐Fisher) for 10 min. Then, cells were blocked in an immunofluorescence (IF) blocking buffer (12411S, Cell Signalling Technology) for 30 min at RT and further incubated with primary antibodies at 4°C overnight. The primary antibodies used include lamin A/C (ab133256, Abcam) (dilution 1:100), activated YAP1 (ab205270, Abcam) (dilution 1:500), SUN1 (ab103021, Abcam) (dilution 1:200), SUN2 (ab124916, Abcam) (dilution 1:200) and phosphorylated H2A histone family member X (γH2AX) (ab81299, Abcam) (dilution 1:250). The secondary antibodies that were used included goat anti‐rabbit IgG H&L Alex Fluor 594 (ab150080, Abcam) or Alexa Fluor 488(ab150077, Abcam). After washing with PBS, samples were incubated with a phalloidin‐iFluor 488 Reagent (ab176753, Abcam). After rinsing with PBS 3 times, all samples were mounted using a fluorescent mounting medium with DAPI (ab104139, Abcam). The staining was analysed using a confocal laser microscope (LSM980 Airyscan 2, Carl Zeiss).

### 2.14 Microscope Parameters

All fluorescent images were captured using either an inverted phase contrast fluorescence microscope (DMI6000 B, Leica) with HCX PL FLUOTAR 10×/0.3 and 63×/0.7 dipping lenses or a confocal laser microscope (LSM980 Airyscan 2, Carl Zeiss) equipped with a Plan Apo 63×/1.4 oil immersion objective. Images were acquired with hybrid detectors configured to the following detection ranges: green, 491–646 nm; red/pink, 578–757 nm; and blue, 426–544 nm. Excitation was provided by a filtered white light source at 493 and 590 nm and a UV laser at 353 nm.

### 2.15 Nuclear‐Cytoplasmic Fractionation Experiment

Cells were grown to approximately 80% confluency and harvested by scratching. The cells were collected by centrifugation at 300 × g for 5 min at 4°C. The supernatant was discarded, and the cell pellet was resuspended in lysis buffer (50 mM tris–HCl (pH 7.5), 150 mM NaCl, 1 mM EDTA, 1% Trition X‐100 and protease inhibitors). The lysate was centrifuged at 600× *g* for 10 min at 4°C. The nuclear pellet was resuspended in RIPA buffer supplemented with protease inhibitors. The supernatant, containing the cytoplasmic fraction, was carefully collected and mixed with an equal volume of RIPA buffer. Both of them are incubated on ice for 30 min and stored at −80°C for further analysis.

### 2.16 Statistical Analysis

Statistical analyses were performed using the GraphPad Prism 9.0 software (GraphPad software, La Jolla, CA, USA). All values are expressed as means ± standard deviation (SD) and were analysed using one‐way ANOVA or Student's *t*‐test to determine the statistically significant differences between groups. Differences were considered statistically significant at a *p* value of < 0.05. All experiments were successfully conducted with at least three biological replicates.

## 3 Results

### 3.1 Compression Force–Mediated Shrunken Nuclei, Heterochromatin Formation and Proliferation Inhibition

**FIGURE 2:**
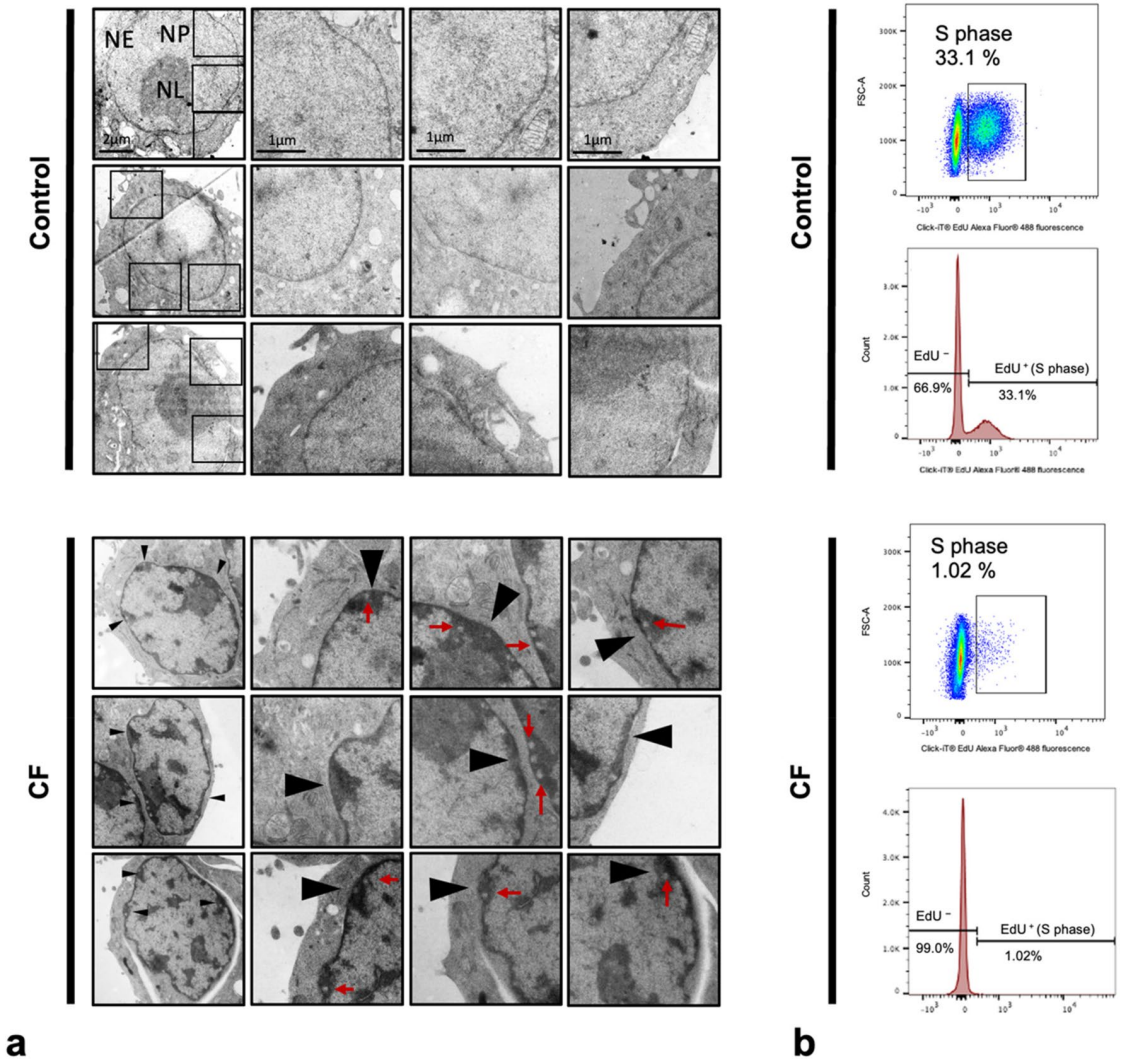
Compressive force induced heterochromatin formation and inhibited proliferation. (a) Transmission electron microscopy (TEM) images showed increased heterochromatin formation within the nucleus after 24 h, localised along the nuclear envelope. Vesicle‐like structures were also observed at the sites of interaction between heterochromatin and the nuclear envelope; (b) compressive force significantly reduced the proportion of cells in the S‐phase. black arrows, heterochromatin; CF, compressive force; NE, nuclear envelope; NL, nucleolus; NP, nucleoplasm; red arrows, vesicles.

The macrophages were subjected to a static compressive force of 1 g/cm^2^ for 24 h. We observed a substantial increase in shrunken nuclei, yet their nuclear envelopes retained their intact structures (Figure 2a). Additionally, a significant increase in heterochromatin was observed within these cells, tightly adhering to the nuclear envelope (Figure 2a, black arrows). Moreover, we observed vesicle‐like structures at the region of heterochromatin (Figure 2a, red arrows), with still unknown specific composition and biological role. These findings support the presumption that chromatin plays a role in mechanotransduction by condensing itself. More importantly, the subnuclear localisation of these condensed heterochromatin suggests that the nuclear envelope also responds to compressive force by interacting with heterochromatin. Given the observed vesicle‐like structures, we think the response of the nuclear envelope is likely associated with increased nuclear‐cytoplasmic transport. Hence, the responses of the nuclear membrane and its associated structures under compressive force serve as the focus of this study.

Additionally, we assessed the impact of compressive force on the proliferation of macrophages using an EdU incorporation assay. Cells undergoing proliferation incorporate EdU into their DNA and can be detected as EdU‐positive cells by flow cytometry. After 24 h of compressive force stimulation, we observed almost no S‐phase cells, indicating that compressive force significantly inhibits cell proliferation (Figure 2b).

### 3.2 Compressive Force–Induced Transient Lamin A/C Deficiency

**FIGURE 3:**
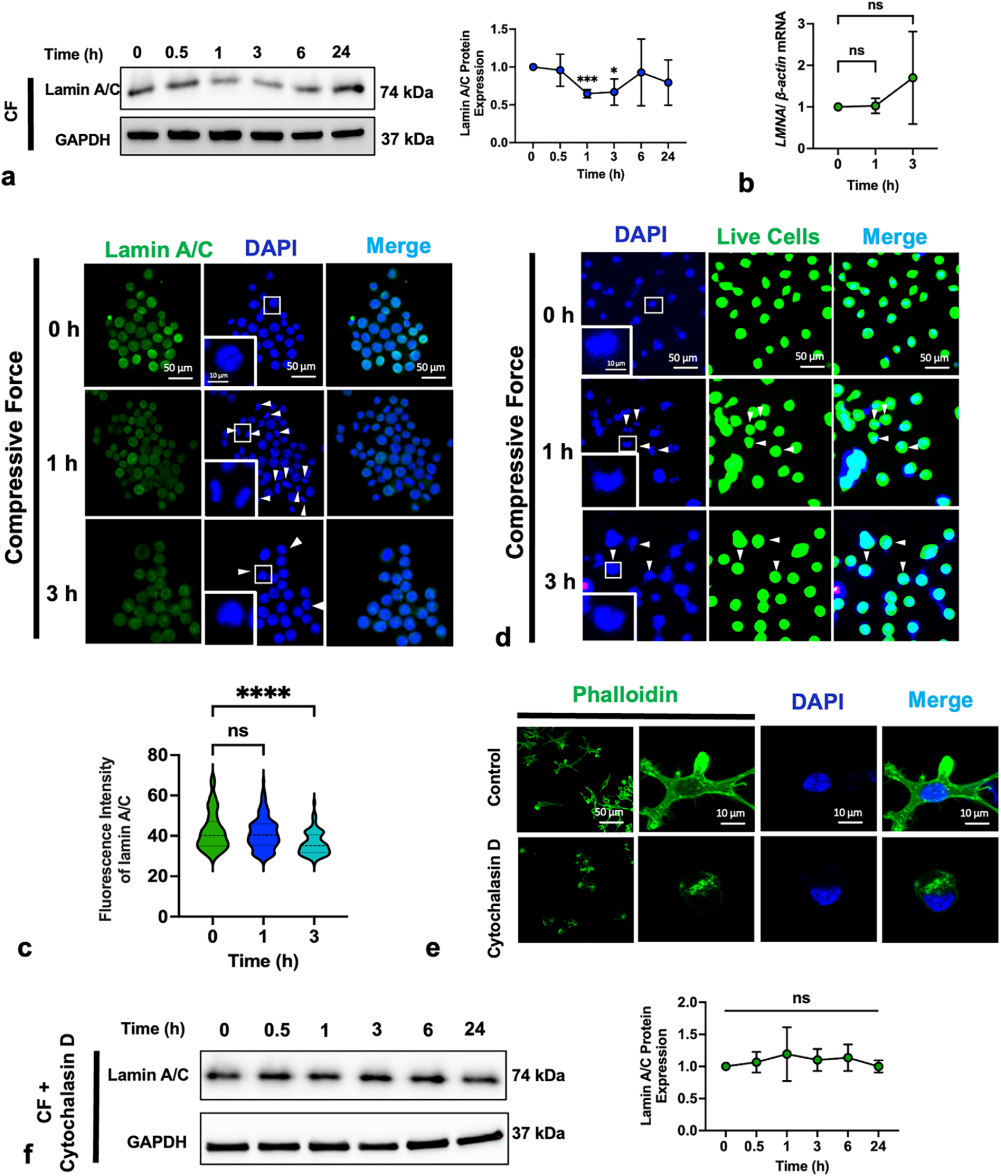
Compressive force induced cytoskeleton‐dependent lamin A/C protein suppression. (a, b) Compressive force transiently downregulated lamin A/C at the protein level after 1 and 3 h; (c) immunofluorescent staining and quantification demonstrated a transient decrease in lamin A/C and deformed nuclei induced by compression (white arrows indicate morphologically abnormal nuclei); (d) Calcein AM staining confirmed that cells with shrunken nuclei remained viable; (e) cytochalasin D caused cytoskeleton destruction; (f) cytochalasin D eliminated the inhibition of lamin A/C by compressive force. CF: compressive force. Values are expressed as means ± SD: Ns—not significant; *p < 0.05; ***p < 0.001; and ****p < 0.0001.

Lamin A/C constitutes a protein meshwork located beneath the nuclear envelope. After applying compressive force, we observed a significant decrease in the lamin A/C expression at 1 and 3 h, with reductions of 35.25% at 1 h and 33.11% at 3 h. Notably, lamin A/C levels returned to baseline by the 6‐h mark (Figure 3a). Next, we stained the macrophages with an anti‐lamin A/C primary antibody and a fluorescent secondary antibody and then observed them using a confocal laser fluorescence microscopy. The fluorescence intensity of lamin A/C exhibited a significant decrease at 3 h (Figure 3c). However, there was no alteration in gene expression levels at either 1 or 3 h (Figure 3b). These findings suggest that compressive force induces a transient lamin A/C deficiency at the protein level rather than the gene level.

Moreover, a considerable proportion of macrophage nuclei displayed abnormal morphology at 1 h, resembling shrunken peanut‐like structures, which then partially recovered at 3 h (Figure 3c,d). These macrophages with deformed nuclei still maintained a normal cellular profile and viability according to Calcein AM staining (Figure 3d). Interestingly, this nuclear deformation and lamin A/C protein reduction were both transient and essentially synchronised, being most prominent at 1 h and then diminishing over time (Figure 3a,c). These dynamic alterations suggest that compressive force can condense nuclear structures; however, the nuclei gradually adapt to the compressive force, partially recovering to their original structure.

To further elucidate the association between compressive force and the reduction of lamin A/C, we disrupted the cytoskeleton to prevent force transmission. Macrophages were pre‐incubated with 2 μM cytochalasin D (PHZ1063, ThermoFisher) for 24 h, a cytoskeleton‐disrupting agent that induces F‐actin depolymerisation [31, 32], before being subjected to compressive force. The control group was treated with an equivalent volume of DMSO prior to compression force stimulation. Phalloidin staining revealed significant cytoskeletal disruption (Figure 3e), and Western blot analysis showed that lamin A/C was unable to respond to compressive force at all time points (Figure 3f), suggesting that the force‐induced lamin A/C deficiency is cytoskeleton‐dependent.

### 3.3 Lamin A/C Deficiency Increased Nuclear Permeability

**FIGURE 4:**
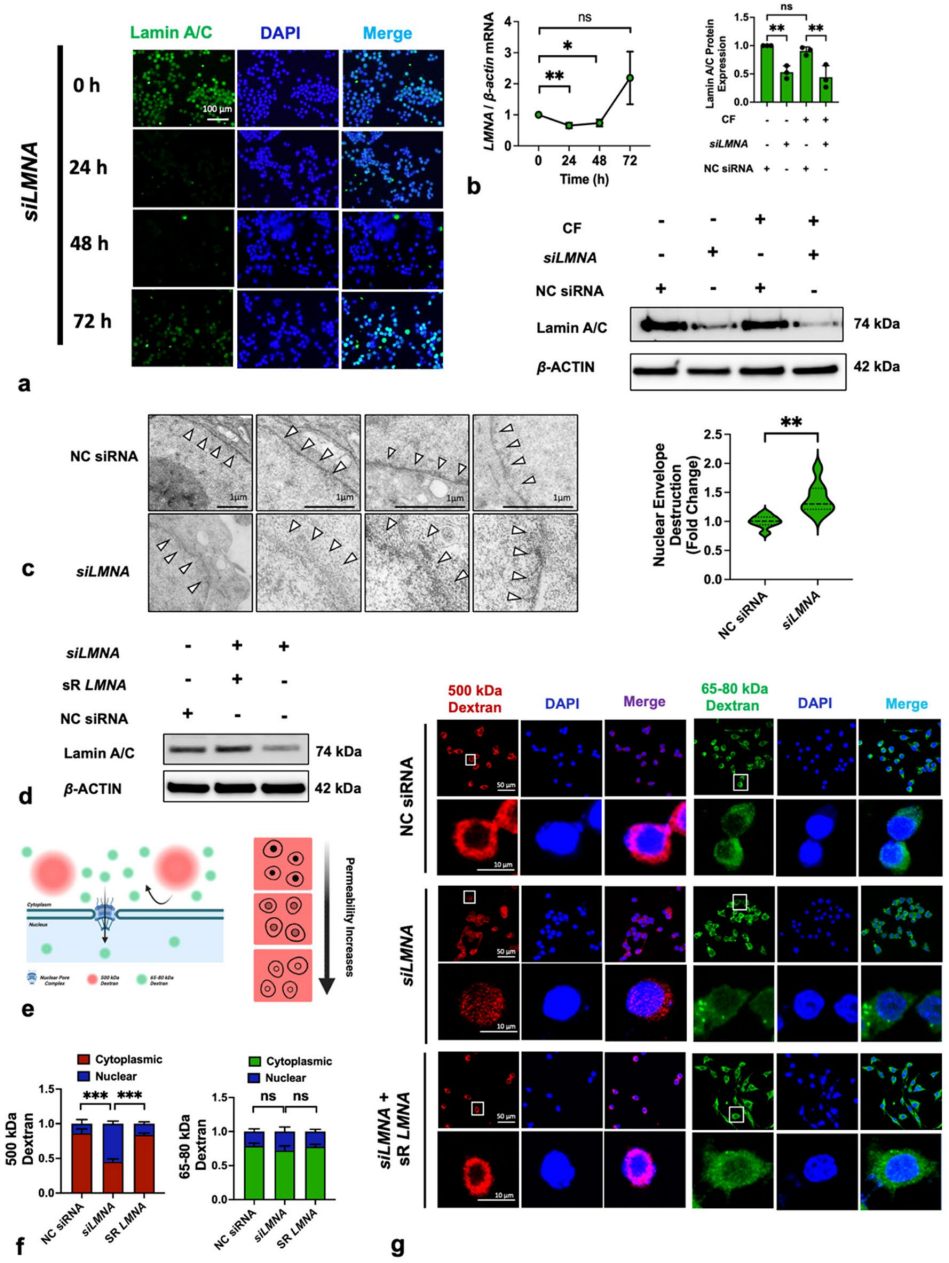
*siLMNA* induced lamin A/C deficiency and enhanced nuclear permeability. (a) IF results showed that *siLMNA* downregulated lamin A/C protein levels; (b, c) *siLMNA* suppressed the *LMNA* gene expression during the 24‐ to 48‐h period and reduced lamin A/C protein levels, whether compressive force was present or not; (d) TEM displayed that *siLMNA* caused impairment of the nuclear envelope; (e) under physiological conditions, 65–85‐kDa dextran can passively transport into the nucleus through NPC but 500‐kDa dextran cannot permeate the nuclear envelope due to its larger molecular diameter than NPCs. A schematic graphic exhibited the cells with different permeabilities to dextran; (f, g) the *siLMNA* significantly increased the nuclear permeability to 500‐kDa dextran and sR *LMNA* eliminated the effect. CF, compressive force; NC, negative control; ns, not significant. White arrows: nuclear envelope. Values are expressed as means ± SD. *p < 0.05; **p < 0.01; and ***p < 0.001.

To further investigate the role of lamin A/C, we transfected macrophages with siRNA to achieve a more sustained lamin A/C deficiency. qRT‐PCR analysis revealed a significant decrease in *LMNA*‐mRNA after 24 and 48 h (Figure 4b). Consistently, IF staining showed that lamin A/C protein levels decreased at 24‐ and 48‐h post‐transfection but returned to baseline levels after 72 h (Figure 4a). Western blotting results demonstrated that at 48 h post‐transfection, the lamin A/C protein inhibition rate reached approximately 44%–52% (Figure 4b). Hence, for all subsequent studies, we selected 48 h as the transfection duration. Additionally, a 24‐h compressive force loading, which our previous research [15] has verified significantly, affects inflammatory cytokine secretion but did not impact the inhibition of lamin A/C by *siLMNA* (Figure 4b). Lamin A/C is a primary component of the nuclear lamina, a protein network that supports the inner nuclear envelope and encases the nuclear chromatin. The TEM assay displayed that lamin A/C deficiency caused an ambiguous nuclear envelope (Figure 4c). Hence, we hypothesised that lamin A/C deficiency might lead to increased nuclear permeability.

Additionally, we introduced a siRNA‐resistant *LMNA* mutant (sR *LMNA*) to rescue the lamin A/C deficiency induced by *siLMNA*. The results of a protein assay suggested that sR *LMNA* significantly reversed the lamin A/C deficiency at 48 h (Figure 4d). Moreover, we assessed nuclear permeability using two types of fluorescent dextran with different molecular masses. The smaller molecular mass dextran (65–80 kDa) can passively diffuse into the nucleus through the nuclear pore transport complex (NPC), whereas the larger molecular mass dextran (500 kDa) requires NPC deformation or nuclear damage for permeation (Figure 4e). Lamin A/C deficiency significantly increased the permeability of the large molecular dextran (*p* < 0.001). Meanwhile, this increased permeability can be reversed by sR *LMNA* (Figure 4f,g). This evidence confirmed that lamin A/C plays a crucial role in maintaining nuclear isolation of macromolecules.

### 3.4 Lamin A/C Deficiency Enhanced Effects of Compressive Force

**FIGURE 5:**
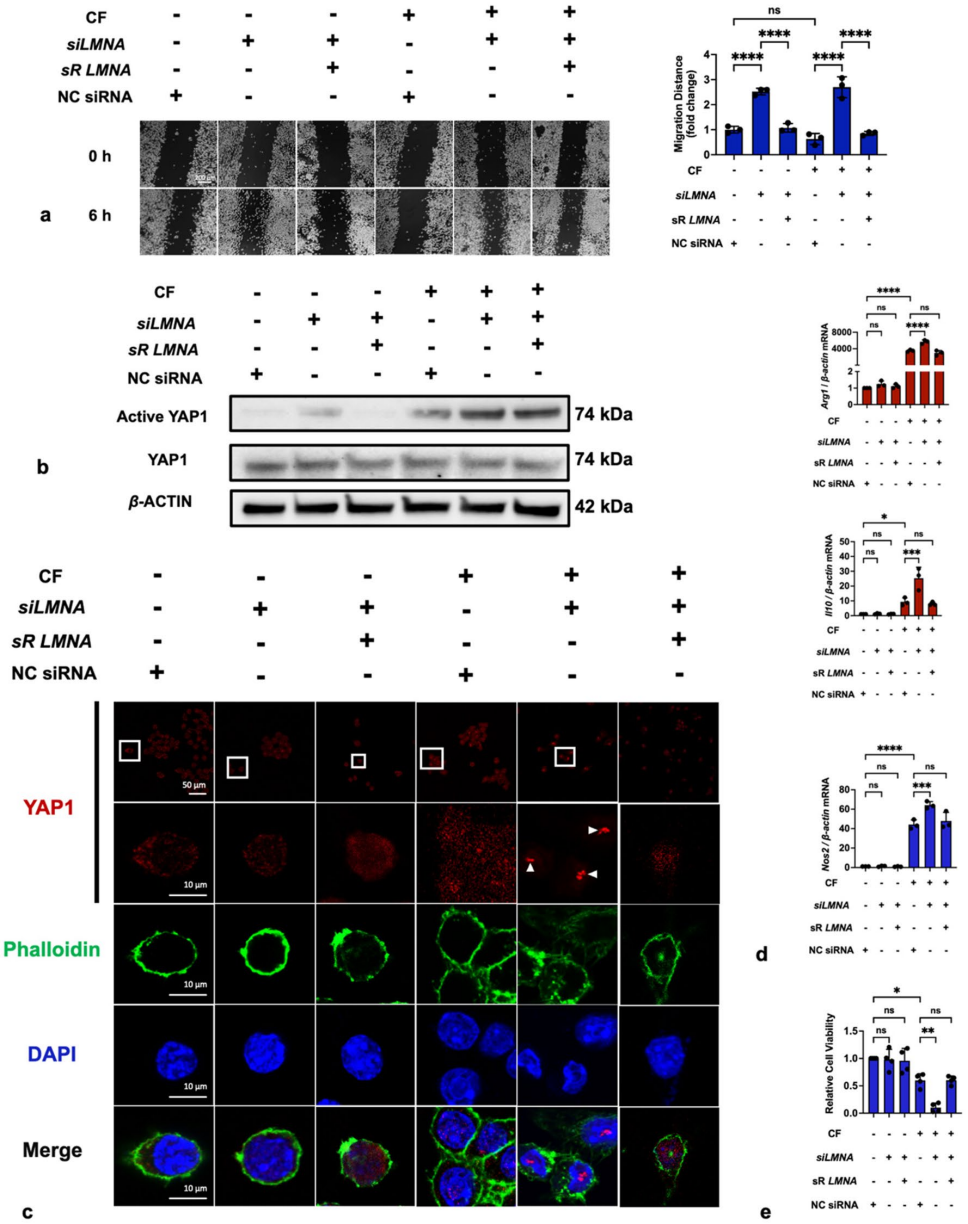
Lamin A/C deficiency enhanced the effects of compressive force, including promoting YAP1 translocation, inducing polarisation and inhibiting proliferation. (a) Lamin A/C deficiency enhanced migration function of macrophages. (b) Lamin A/C deficiency and compressive force both increased active YAP1 protein levels. (c) IF staining showed the distributions of YAP1 in nuclei and cytoplasm (white arrows: YAP1 accumulation in nuclei). (d) Compressive force induced the expression of *Arg1*, *Il10* and *Nos2*, and *siLMNA* enhanced these effects. (e) Compressive force inhibited cellular viability, with *siLMNA* further reducing it. CF, compressive force; NC, negative control; ns, not significant. Values are expressed as means ± SD: *p < 0.05; **p < 0.01; ***p < 0.001; and ****p < 0.0001.

We further investigated the role of lamin A/C in cellular mechanotransduction. First, we evaluated the effects of compressive force on migration. Interestingly, while lamin A/C deficiency induced by siRNA significantly enhanced cellular migration, cells exposed to compressive force, which also induces lamin A/C deficiency, did not show impaired migration (Figure 5a). We hypothesise that while lamin A/C plays an essential role in macrophage migration, the compressive force may introduce other factors related to migration that mask the inhibitory effects typically associated with lamin A/C deficiency.

Second, given the increased nuclear permeability, we hypothesised that lamin A/C deficiency may influence the nuclear translocation of transcription factors involved in mechanotransduction. Yes‐associated protein 1 (YAP1), a well‐known mechanosensitive transcription factor, accumulates in the nucleus under mechanical force [33]. Western blot analysis revealed that both 48‐h *siLMNA* transfection and 24‐h compressive force loading increased the level of active YAP1, instead of total YAP1 (Figure 5b). Moreover, IF demonstrated that the intranuclear signal in the CF + *siLMNA* group was significantly increased compared to other groups (Figure 5c), supporting the hypothesis that lamin A/C restricts compressive force‐induced YAP1 translocation. Another noteworthy observation is that cells subjected to compressive force displayed nuclei that appeared to be shrinking, and cells treated with both compressive force and *siLMNA* exhibited even more pronounced nuclear deformations (Figure 5c). Therefore, it is reasonable to conclude that the nucleus tends to undergo morphological changes under compression, and lamin A/C serves as a structural support.

Moreover, YAP1 is implicated in regulating cellular proliferation [34] and macrophage polarisation [35]. Thus, we further examined the gene expressions of *Arg1*, *Il10* and *NOS2*, as well as proliferation. As expected, a 1‐g/cm^2^ compression elicited inflammatory gene expression and inhibited cell viability, with deficient lamin A/C exacerbating these effects (Figure 5d,e). Interestingly, while comparing the CF + *siLMNA* group with the CF group, *siLMNA* promoted the nuclear localisation of YAP1 rather than its protein level; however, it still intensified the effects of compressive force on polarisation markers and proliferative viability (Figure 5d,e). These suggest that the subcellular localisation of YAP1 may be more critical than its total level in mechanotransduction. Furthermore, as YAP1 concentration increased in the nucleus, the signal tended to form punctate aggregations. This could suggest the presence of regions within the nucleus with a high affinity for YAP1, such as nucleoli, nuclear bodies or specific chromatin sites, where YAP1 preferentially accumulates rather than being uniformly distributed. However, more evidence is needed to definitively identify these high‐affinity regions or substances. In conclusion, lamin A/C limits the cellular response to compressive force by restricting the nuclear translocation of YAP1.

### 3.5 Lamin A/C Deficiency Detached LINC Complex From Nucleus

**FIGURE 6:**
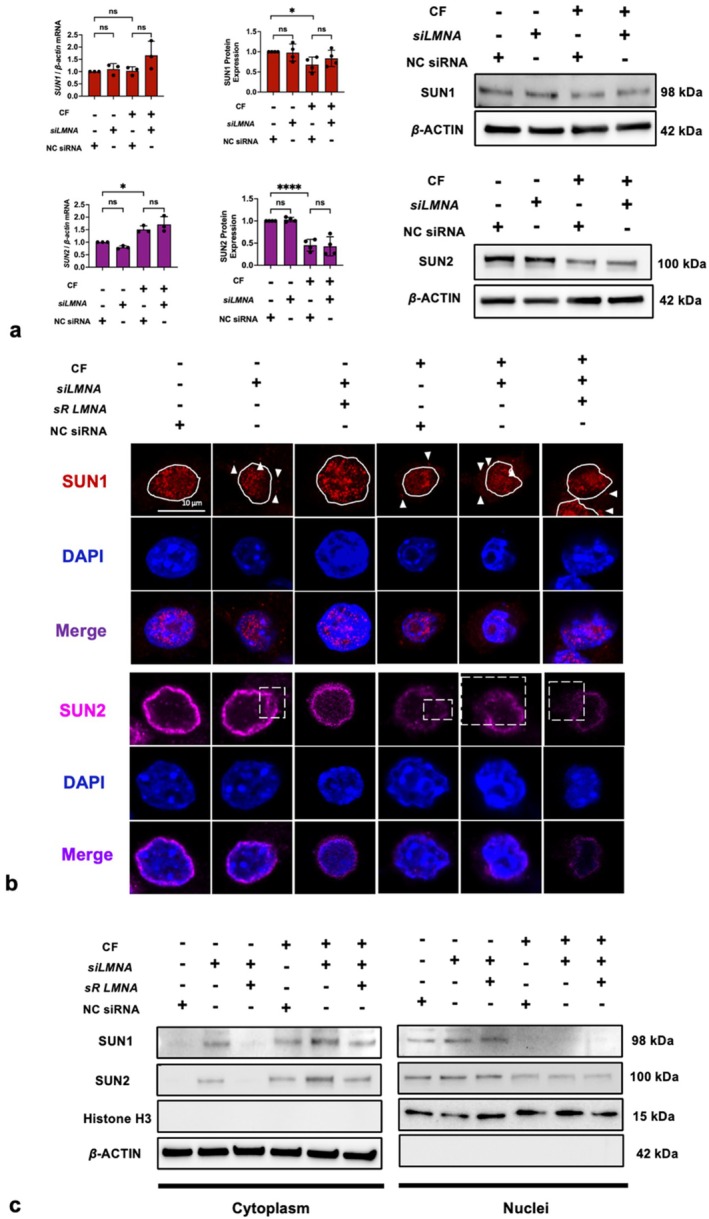
Compressive force and *siLMNA* influenced the LINC complex. (a—d) Compressive force, instead of siLMNA, significantly decreased the protein expression of SUN1 and SUN2; (g, h) compressive force or (and) *siLMNA* detached SUN1 and SUN2 from the nuclei (white solid line: the boundaries of the cell nucleus; white arrows: SUN1 signals in cytoplasm; white square: discontinuous signal indicating SUN2 destruction). CF, compressive force; NC, negative control; ns, not significant. Values are expressed as means ± SD. *p < 0.05; and ****p < 0.0001.

The LINC complex is a vital structure that connects the cytoskeleton to the lamina, playing a critical role in intracellular mechanotransmission [36]. Therefore, we evaluated the impacts of lamin A/C deficiency and compressive force on the LINC complex. The protein levels of SUN1 and SUN2, components of LINC complex, were significantly suppressed by compressive force but unaffected by *siLMNA* (Figure 6a). Moreover, based on IF images, SUN1 signals are assembled in the nucleus, but *siLMNA* or (and) compressive force caused SUN1 to diffuse into the cytoplasm (Figure 6b). Similarly, an intact circle of SUN2 that surrounded the nucleus was observed in the control group but *siLMNA* or (and) compressive force resulted in the distinct disruption of the SUN2 circle (Figure 6b). Notably, these detachments of SUN1 and SUN2 were reversed by the introduction of sR *LMNA*. Additionally, we conducted a fractionation experiment to separate nuclear and cytoplasmic components. Through centrifugation, the nuclear envelope, nucleolus, chromatin and other nuclear materials are separated into the nuclear fraction, while cell membrane fragments, cytoplasm, endoplasmic reticulum, mitochondria and other components are separated into the cytoplasmic fraction. SUN1 and SUN2 were detected in the cytoplasmic fraction whenever lamin A/C deficiency or compressive force was applied (Figure 6c). In summary, only compressive force reduced the protein SUN1 and SUN2, but both compressive force and lamin A/C deficiency led to the detachment of SUN1 and SUN2 from the nucleus.

### 3.6 Lamin A/C Deficiency Inhibited Compressive Force–Induced DNA Damage and IRF4 Expression

**FIGURE 7:**
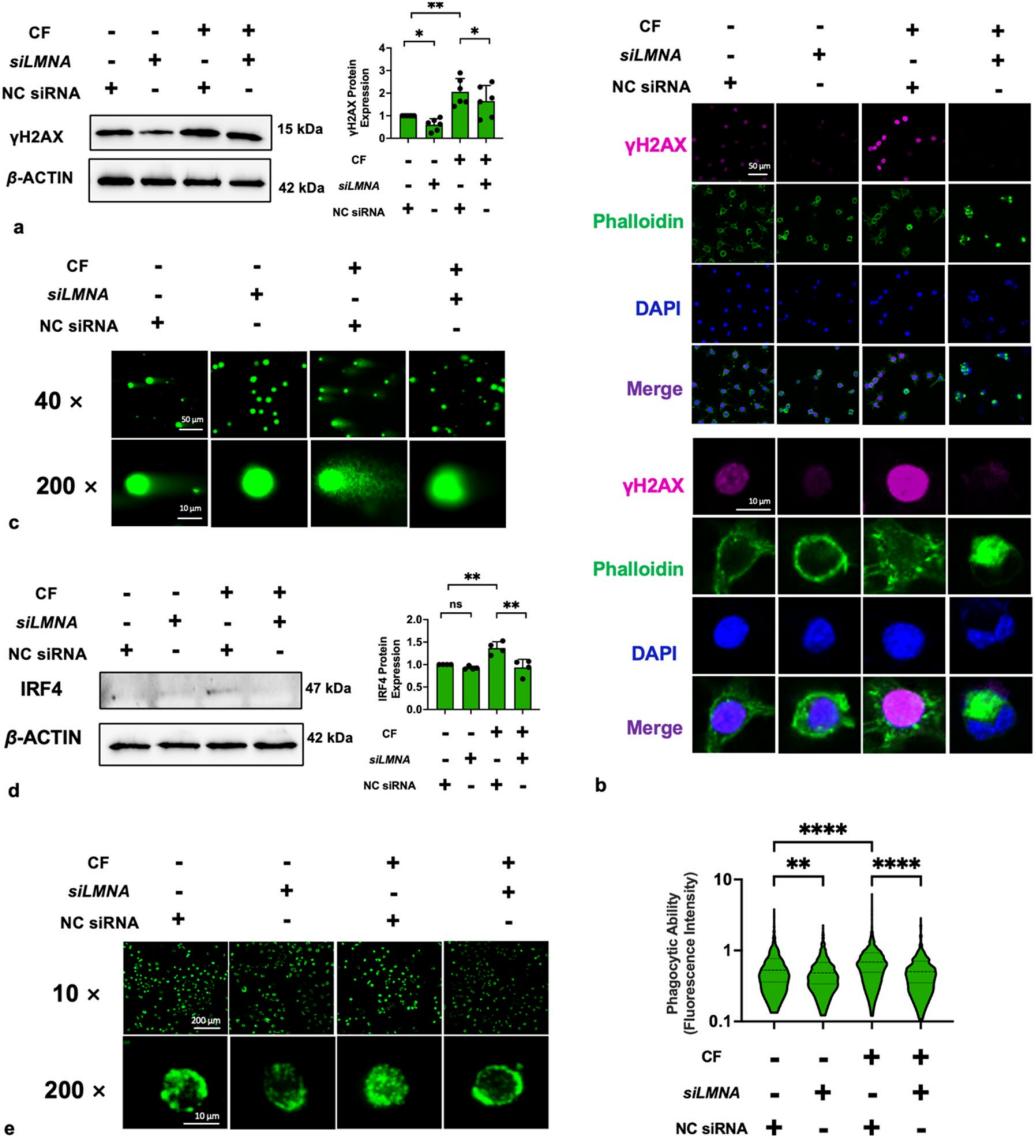
Compressive force–induced DNA damage was inhibited by *siLMNA*. (a, b) The effects of compressive force or (and) *siLMNA* on γ‐H2AX; (c) Comet assay revealed that compressive force caused more DNA fragments, but *siLMNA* eliminated this effect; (d) the effects of compressive force or (and) *siLMNA* on IRF4 protein level; and (e) the macrophage phagocytosis function was enhanced by compressive force but inhibited by *siLMNA*. CF, compressive force; NC, negative control; ns, not significant. Values are expressed as means ± SD. *p < 0.05; **p < 0.01; ***p < 0.001; and ****p < 0.0001.

Considering that both compressive force and *siLMNA* resulted in nuclear deformation, we assessed their effects on DNA damage. γH2AX, a biomarker for DNA double‐strand breaks [37], was assayed by IF and Western blot. These experiments revealed that γH2AX is located in nuclei and is significantly increased by compressive force (Figure 7a,b). Interestingly, although lamin A/C is known as a nuclear supporter due to its proportional relationship with nuclear stiffness, *siLMNA* reduced significantly γH2AX protein levels (Figure 7a). We further corroborated these findings with a comet assay. Specifically, cells subjected to compressive force had the longest ‘comet tail’ comprised of DNA fragments and *siLMNA* significantly shortened these tails (Figure 7c). Therefore, we believed that compressive force elicited DNA damage in macrophages, and this damage was reduced by lamin A/C deficiency. Consistently, our results also demonstrated that compressive force increased interferon regulatory factor 4 (IRF4) levels, a transcription factor associated with the anti‐inflammatory activation of macrophages [38, 39], and phagocytosis. Notably, these two effects can be eliminated by lamin A/C deficiency (Figure 7d,e). These results showed that lamin A/C deficiency can reverse some effects of compressive force on macrophages (promoting DNA damage, IRF4 expression and phagocytosis), rather than enhancing them.

## 4 Discussion

Cellular mechanosensation is involved in an extensive range of pathological and physiological processes, including nerve impulses [40], innate immunity [41], tissue morphogenesis [42, 43], wound healing [44], skin haemostasis [45], bone remodelling [46, 47], orthodontic tooth movement [48], cancer progression [49, 50], periodontitis [51], pulmonary fibrosis [52], portal hypertension [53] and atherosclerosis [54]. Therefore, it is critically important to understand the mechanisms of cellular mechanosensation. Beyond the well‐known mechanoreceptors on the cell membrane surface (such as Piezo1/2 and TRPV4), nuclei can also respond to mechanical force [13, 55]. Our data have identified the significant role that lamin A/C plays in the macrophage nuclear response to static compressive force.

Our data showed that macrophage nuclei responded to compression by forming heterochromatin (Figure 2a). Stephens et al. [56] observed a similar phenomenon in mesenchymal stem cells. These highly condensed chromatin structures were believed to increase nuclear stiffness against compression, thereby reducing nuclear rupture and DNA damage. Meanwhile, heterochromatin has a lower transcriptional activity; therefore, the condensation of this chromatin may be one of the mechanisms by which mechanical forces regulate gene transcription [57].

Interestingly, we observed that cells exhibiting excessive heterochromatin formation showed a lower proportion of S‐phase, indicating a reduced proliferative viability (Figure 2b). This was also supported by our related viability assay (Figure 5e). The stabilisation of heterochromatin has been widely reported to be associated with cellular aging and proliferation arrest [58, 59, 60, 61]. However, a limitation of our study is the inability to confirm a direct causal relationship between mechanically induced heterochromatin condensation and inhibition of proliferation.

Another interesting observation is that some heterochromatin was co‐localised with the inner nuclear envelope and vesicles. It remains unclear why these condensed chromatins adhere to the nuclear envelope. However, the ‘vesicle‐like’ structures observed within the heterochromatin offer a potential explanation; this adhesion may facilitate the exchange of heterochromatin components with the extranuclear environment.

Further, we found that the compression inhibited the lamin A/C level (Figure 3a,c), aligning with the results of the studies by Chambliss et al. [62] and Fu et al. [63]. Interestingly, in different cell lines, lamin A/C seem to respond differently, or even oppositely, to mechanical force. Maremonti et al. [64] observed contrasting responses in two different human epithelial cell lines (MDA‐MB‐231 and MCF‐10A) subjected to the same mechanical stimulation, with an increase in MCF‐10A cells but a decrease in MDA‐MB‐231 cells. Anyway, our study demonstrated that macrophages (RAW264.7) responded to compressive force via reducing lamin A/C, and this reduction was cytoskeleton‐dependent (Figure 3f). It suggests that force is transmitted along the cytoskeleton to reach the lamina.

Moreover, this force‐induced lamin A/C deficiency was not constant. Western blotting analysis showed that lamin A/C levels were inhibited after 1 h but completely recovered after 6 h (Figure 3a). Lamin A/C deficiency induced by *siRNA* was also recovered after 72 h at both the genetic and protein levels (Figure 4a,b). Considering that lamin A undergoes periodic dynamic changes throughout the cell cycle [20, 65, 66], we suggest that the cell has a lamin A/C repair mechanism. Specifically, compressive force impairs lamin A/C in the early stage, and a repair mechanism is subsequently initiated to rescue the lamin A/C level and to re‐strengthen nuclear stiffness for resistance against external pressure. It was reported that the *LMNA*‐mRNA expression level depends on the existing amount of *LMNA*‐related proteins [23], providing further support for our repair mechanism theory. This theory could explain first why the macrophages respond differently to compressive force over time since the repair mechanism following the initial cellular response causes the dynamic alteration of the cellular mechanical effector (lamin A/C). It explains second why distinct cell lines exhibit contrasting responses of lamin A/C to the same mechanical stimulation. We propose that the repair mechanism may overcompensate in those upregulation cases.

The role of lamin A/C on cells in mechanotransduction is the focus of this study. At first, lamin A/C levels determine nuclear rigidity [67]. After 1 h of force loading, lamin A/C decreased and nuclear deformation occurred concurrently (Figure 3c,d). Meanwhile, *LMNA* knockdown enhanced compressive force–induced nuclear deformation (Figures 5c and 6b). These findings align with observations that fibroblasts that lack lamin A/C face an increased risk of nuclear rupture under compression [68, 69], supporting the conventional theory that lamin A/C, serving as the nuclear scaffold, counteracts mechanical pressure's impact on the nucleus [70, 71].

Due to the transient nature of force‐induced lamin A/C deficiency, we employed *siRNA* to induce lamin A/C deficiency for longer periods to conduct other gene and protein analyses. *LMNA* was silenced from post‐transfection 24–48 h (Figure 4a–c), but lamin A/C protein level could be completely rescued by sR *LMNA* after 48 h (Figure 4d).

The lamin A/C deficiency caused nuclear envelope impairment and increased nuclear permeability (Figure 4f,g). This increased permeability coincided with enhanced nuclear translocation of YAP1 (Figure 5c). YAP1 is a classic mechanical response protein. Numerous studies have shown that it mediates the mechanotransduction by translocating into the cellular nucleus [72, 73, 74, 75, 76, 77]. YAP1 can modulate cellular inflammation [35] and proliferation [78]. Thus, we detected expressions of inflammatory genes (*Arg1*, *Il10* and *Nos2*) and the cellular proliferative ability using qRT‐PCR and viability assay (Figure 5d,e). The results showed that deficient lamin A/C levels significantly enhanced the effects of force on macrophage, including the induction of polarisation and inhibition of proliferation. These results suggested that deficient lamin A/C plays a positive role in mechanotransduction by increasing the nuclear permeability and YAP1 translocation.

**FIGURE 8:**
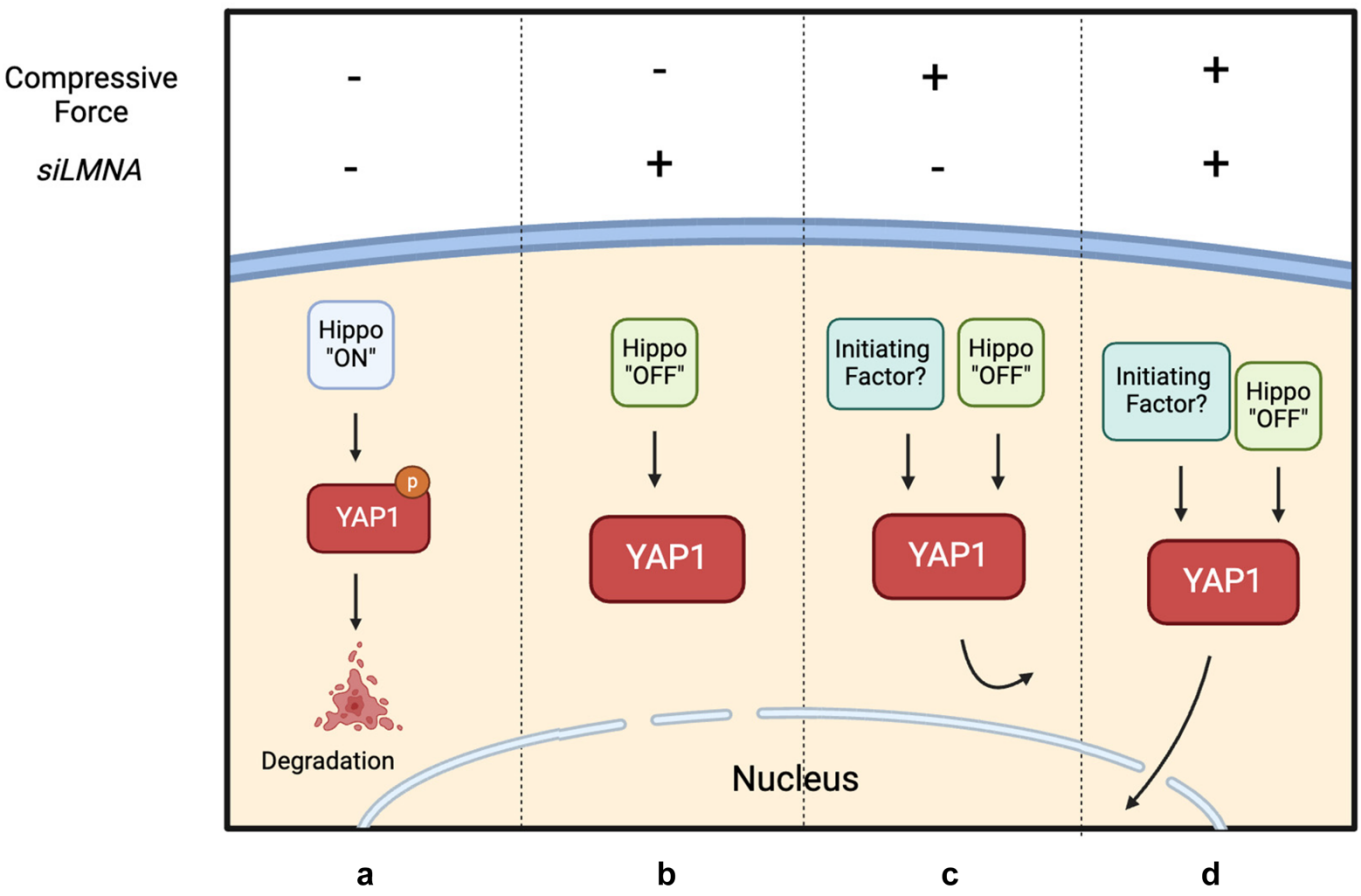
A simplified schematic diagram illustrates the regulation of YAP1 translocation in four different states: (a) In the physiological state, the Hippo pathway phosphorylates (inactivates) YAP1, leading to its degradation in the cytoplasm. (b) Although siLMNA interferes with YAP1 phosphorylation, YAP1 cannot translocate into the nucleus due to the absence of the ‘initial factor’ associated with compressive force. (c) Due to the mechanical impedance of lamin A/C, activated YAP1 accumulates in the cytoplasm. (d) Compressive force drives the activated YAP1 to translocate into the nucleus, overcoming the deficiency in lamin A/C.

Interestingly, apart from limiting the nuclear translocation of YAP1, lamin A/C could also regulate its protein level. The classical regulatory mechanism of YAP1 is the Hippo signalling pathway [79]. Briefly, the Hippo signalling pathway is initiated when the large tumour suppressor kinase 1/2 (LATS1/2) complex is phosphorylated, leading to the downstream phosphorylation of YAP1. Phosphorylated YAP1 binds to 14‐3‐3 proteins and is eventually degraded in the cytoplasm. When the Hippo signalling pathway is ‘turned off’, non‐phosphorylated YAP1 translocates into the nucleus and triggers gene transcription. However, what cannot be explained by the classical modulation theory is that, in our experiments, although either force or lamin A/C deficiency can upregulate YAP1 levels, it does not increase its nuclear translocation. Only under the combined action of compressive force and lamin A/C deficiency that significant nuclear translocation of YAP1 occurs (Figure 5c). Thus, we proposed a model to explain regulated mechanisms of YAP1 translocation by compressive force and lamin A/C (Figure 8). Specifically, compressive force provided an unknown initiating factor, and deficient lamin A/C levels induced nuclear envelope impairment to permit translocation. There are many possible explanations for the exact nature of this unknown initiation signal. Here are two hypotheses we propose: a. compressive force probably stretches nuclear pores, enhancing YAP1 nuclear translocation [73, 80]; b. mechanical clues may be necessary for the recognition of importin, a nuclear transport carrier, to recognise the nuclear localisation signal (NLS) of YAP1. However, a more definitive conclusion cannot be drawn until more relevant research results are revealed.

Another significant effect of lamin A/C deficiency is its role in regulating migration. Knockdown of lamin A/C via *siLMNA* significantly enhances macrophage migration (Figure 5a). In mesenchymal stem cells, reduced levels of lamin A/C contribute to a softer nuclear structure, thereby facilitating migration [17]. A similar phenomenon has been observed in breast cancer cells [68], aligning with our data. However, while compressive force can also decrease lamin A/C levels, it fails to promote cell migration. We hypothesise that this may be due to compressive forces triggering other factors that counteract migration, such as hypoxia.

On the other hand, we also found that lamin A/C deficiency has a negative impact on mechanotransduction. We observed that lamin A/C deficiency induced detachment of the LINC complex from the nuclei (Figure 6b,c). The LINC complex forms a physical connection between cytoskeleton and nucleoskeleton, which transduces the mechanical signals from the environment to the nuclei [27]. The LINC complex consists of an outer nuclear membrane, the KASH domain protein and an INM SUN domain protein. SUN1 and SUN2 constitute the INM component of the LINC complex, interacting with lamina and connecting with the KASH domains of nesprins in the perinuclear space (PNS) [69, 81]. Some scholars have reported that the deletion of lamin A/C impairs the nuclear localisation of SUN2 [82, 83], which is consistent with our findings, whereas its effects on SUN1 is controversial. Chiarini et al. [84] and Mattioli et al. [85] reported that the presence of lamin A/C is significantly proportional to the nuclear localisation of SUN1, but Haque et al. [83] reported that lamin A/C did not affect SUN1 localisation and believed that SUN1 was anchored on the nucleus by binding to chromatin rather than the lamina. Different conclusions may be due to different cell lines that were used. Anyway, our data clearly demonstrated that deficient lamin A/C levels caused detachment of SUN1 and SUN2 from nuclei, impeding mechanotransduction (Figure 6b,c). Considering that deficient lamin A/C can be caused by compressive force in the early stage, we believe that this negative feedback loop represents a cellular self‐protective mechanism from excessive mechanosensation. A convincing piece of evidence is that some effects of compressive force can be inhibited by lamin A/C deficiency, such as IRF4 expression, DNA damage and enhanced phagocytosis induced by compressive force (Figure 7).

Finally, the unexpected observation that SUN1 and SUN2 were significantly inhibited by compressive force occurred (Figure 6a). The inhibition of SUN2 (57%) is even more significant compared to that of lamin A/C (34%). Gilbert et al. [29] reported that another form of mechanical stimulation, cyclic tensile strain (CTS), can also downregulate SUN2 expression. Three CTS‐sensitive phosphorylation sites on the lamin‐binding domain of SUN2 have been identified, which may induce the rapid loss of SUN2. This SUN2 loss disrupts mechanotransmission between the cytoskeleton and the nucleus, thereby protecting chromatin. On the other hand, evidence suggests that SUN proteins regulate downstream processes such as histone methylation, cytoskeletal metabolism and DNA repair [29, 86, 87]. Therefore, we propose that the SUN proteins may couple mechanical stimulation with these biological processes, not only by serving as a mechanical linker but also through direct interactions with chromatin and the regulation of gene expression.

This study elucidates how lamin A/C influences intracellular mechanotransduction under compressive force, highlighting potential pathways through which mechanical stimuli affect cellular behaviour. Clinically, the findings suggest that lamin A/C plays a crucial role in modulating macrophage functions, such as inflammation and proliferation, in response to mechanical stress, which in turn contributes to disease progression. These insights have significant implications for understanding the mechanisms that underly various mechanotransduction‐related diseases, including hyperventilation pneumonia, hypertension‐related cardiovascular lesions, bone remodelling induced by mechanical loading and tumours within specific microenvironments.

The main limitation of this study is the lack of detailed dynamic visualisation of the internal structure of the nucleus under pressure. Cellular response to compressive force is a dynamic process involving lamina rearrangement, chromatin remodelling and nucleocytoplasmic transport. Understanding how these physiological processes affect cellular behaviour is an intriguing topic. This issue may be addressed with advances in live cell imaging technologies in the future. Additionally, all experiments were conducted using a two‐dimensional pressure model. Cells within human tissues may exhibit different behaviours under three‐dimensional pressure, highlighting the need for more accurate three‐dimensional pressure stimulation models in future research.

## 5 Conclusions

Our study highlighted that compressive force induces chromatin rearrangement and transiently downregulates macrophage lamin A/C levels. This transient downregulation impairs the nuclear envelope and increases nuclear permeability to YAP1, subsequently enhancing force‐induced cytokine expression and proliferative inhibition. Meanwhile, it also reduces force‐induced IRF4 expression, DNA damage and enhancement of phagocytosis, probably due to the disrupted LINC complex. These two downstream effects play antagonistic roles in mechanotransduction, jointly regulating cellular behaviour under pressure.

Overall, understanding the dual roles of lamin A/C in mechanotransduction could pave the way for novel therapeutic strategies. For instance, targeting lamin A/C or its regulatory pathways might allow for the modulation of macrophage responses in inflammatory and fibrotic diseases. Additionally, the insights gained from this study could inform the development of new approaches to control cellular behaviour in tissue engineering and regenerative medicine, where mechanical forces are crucial.

## Author Contributions

**Yao Wang:** conceptualisation (equal), data curation (equal), formal analysis (equal), methodology (equal), software (equal), validation (equal), and writing – original draft (equal). **Sabine Ruf:** investigation (equal), methodology (equal), project administration (equal), validation (equal), writing – review and editing (equal), and funding acquisition (equal). **Lei Wang:** formal analysis (equal) and methodology (equal). **Thomas Heimerl:** data curation (equal) and methodology (equal). **Gert Bange:** data curation (equal) and methodology (equal). **Sabine Groeger:** formal analysis (equal), funding acquisition (equal), methodology (equal), project administration (equal), supervision (equal), validation (equal), and writing – review and editing (equal).

## Ethics Statement

The study did not involve human participants or animal experiment included in the study.

## Conflicts of Interest

The authors declare no conflicts of interest.

## Animal Welfare Considerations

All non‐clinical biomedical experiments followed the guidelines of good laboratory practice (GLP) and the WHO declaration from Helsinki 1964, latest update Seoul 2008 (59th WMA General Assembly, Seoul, October 2008).

## Data Availability

The data that support the findings of this study are available from the corresponding author upon reasonable request.
